# Comprehensive analysis and prognostic assessment of senescence-associated genes in bladder cancer

**DOI:** 10.1007/s12672-024-00987-1

**Published:** 2024-04-26

**Authors:** Ruilin Yang, Jieling He, Wenfeng Luo, Renyang Xiang, Ge Zou, Xintao Zhang, Huang Liu, Junhong Deng

**Affiliations:** 1 Jinan University, 601 Huangpu Avenue West, Tianhe District, Guangzhou, 511400 China; 2 Andrology Clinic, The Affiliated Panyu Central Hospital of Guangzhou Medical University, 8 East Fuyu Road, Qiaonan Street, Panyu District, Guangzhou, 511400 China; 3 Ultrasonography Department, The Affiliated Panyu Central Hospital of Guangzhou Medical University, Guangzhou, 511400 China; 4 Central Laboratory, The Affiliated Panyu Central Hospital of Guangzhou Medical University, Guangzhou, 511400 China; 5 Department of Surgery, The University of HongKong-Shenzhen Hospital, Shenzhen, 518053 Guangdong China; 6 Urology Department, The Affiliated Panyu Central Hospital of Guangzhou Medical University, Guangzhou, 511400 China; 7 Department of Urology, The First Affiliated Hospital of Shenzhen University, Shenzhen Second People’s Hospital, Shenzhen, 511400 China; 8 National Health Commission (NHC) Key Laboratory of Male Reproduction and Genetics, Department of Andrology, Guangdong Provincial Reproductive Science Institute (Guangdong Provincial Fertility Hospital), Human Sperm Bank of Guangdong Province, Guangzhou, China; 9 Department of Andrology, Guangzhou First People’s Hospital, The Second Affiliated Hospital of South China University of Technology, Guangzhou, China

**Keywords:** Bladder cancer, Senescence-associated genes, Transcriptomic analysis, Prognosis, Immunotherapy

## Abstract

**Background:**

The prevalence and mortality of bladder cancer (BLCA) present a significant medical challenge. While the function of senescence-related genes in tumor development is recognized, their prognostic significance in BLCA has not been thoroughly explored.

**Methods:**

BLCA transcriptome datasets were sourced from the TCGA and GEO repositories. Gene groupings were determined through differential gene expression and non-negative matrix factorization (NMF) methodologies. Key senescence-linked genes were isolated using singular and multivariate Cox regression analyses, combined with lasso regression. Validation was undertaken with GEO database information. Predictive models, or nomograms, were developed by merging risk metrics with clinical records, and their efficacy was gauged using ROC curve methodologies. The immune response’s dependency on the risk metric was assessed through the immune phenomenon score (IPS). Additionally, we estimated IC50 metrics for potential chemotherapeutic agents.

**Results:**

Reviewing 406 neoplastic and 19 standard tissue specimens from the TCGA repository facilitated the bifurcation of subjects into two unique clusters (C1 and C2) according to senescence-related gene expression. After a stringent statistical evaluation, a set of ten pivotal genes was discerned and applied for risk stratification. Validity tests for the devised nomograms in forecasting 1, 3, and 5-year survival probabilities for BLCA patients were executed via ROC and calibration plots. IC50 estimations highlighted a heightened responsiveness in the low-risk category to agents like cisplatin, cyclopamine, and sorafenib.

**Conclusions:**

In summation, our research emphasizes the prospective utility of risk assessments rooted in senescence-related gene signatures for enhancing BLCA clinical oversight.

**Supplementary Information:**

The online version contains supplementary material available at 10.1007/s12672-024-00987-1.

## Introduction

Bladder cancer (BLCA) ranks prominently among the most frequently diagnosed malignancies within the urological system, accounting for an estimated 540,000 novel cases on a yearly global scale [1, 2]. The aggressive nature of specific bladder tumor variants, notably muscle-invasive bladder cancer, is characterized by profound invasiveness, leading to a heightened mortality risk associated with this ailment [3, 4]. Within oncological practice, there exists a steadfast commitment to the tenet of prompt disease identification and timely therapeutic interventions [5, 6]. Yet, BLCA often manifests insidiously, eluding early symptomatic detection. Consequently, a significant proportion of patients present at advanced stages, characterized by extensive tumor cell infiltration and migration, which frequently hinders therapeutic efficacy and clouds prognosis. It becomes imperative, therefore, to innovate and integrate new prognostic markers to tailor therapeutic strategies for patients diagnosed at advanced BLCA stages [7, 8].

In this context, our research endeavors to delve into the implications of genes associated with cellular senescence. Defined as an irreversible cessation of the cell cycle over time, cellular senescence embodies a decline in cellular function that is ubiquitous across organisms. Cells in this arrested phase resist proliferative cues while concurrently manifesting DNA aberrations. Such senescence acts as a safeguard, curtailing the propagation of DNA-compromised cells and thus, serving an integral role in tumor suppression [9, 10].

In our study, we conducted a detailed analysis of BLCA patient transcriptome datasets from TCGA and GEO to understand the role of senescence-related genes in the disease’s progression. Leveraging these insights, in conjunction with pertinent clinical markers, we’ve crafted and subsequently validated a prognostic algorithm. In addition, we examined the potential signaling pathways of senescence-associated genes, the differences in immune cell infiltration in the tumor microenvironment, the level of somatic mutations, and the differences in risk scores and sensitivity to various chemotherapy drugs.

## Materials and methods

### Collection of multi-omics data

Pertaining to BLCA studies, we sourced both neoplastic and non-neoplastic bladder tissue datasets from GSE31684 and TCGA–BLCA repositories. After excluding entries lacking comprehensive clinical details, we aggregated 93 malignant samples from the GSE31684 dataset. From the TCGA–BLCA repository, we assimilated an additional 406 tumor samples alongside 19 healthy counterparts. Subsequently, we acquired the transcriptomic signatures of these curated samples. To ensure the accuracy of our gene expression analysis from TCGA and GEO datasets, we implemented crucial normalization and batch effect correction steps. Raw data were first normalized with the DESeq2 package to adjust for library size and composition, enabling proper gene expression comparison. For batch effect correction, we used the Combat algorithm from the sva package, targeting both known and unknown batch variables. A thorough review of prevailing academic publications combined with insights from the Genecards database led us to discern 434 prospective genes associated with cellular senescence (Refer to Table S1). To identify differentially expressed genes (DEGs), criteria were established with a false discovery rate (FDR) below 0.05 and a |log2Fold Change (FC)| value surpassing 1.

### Non-negative matrix factorization (NMF) method for tumor subtyping

Tumor classification was executed via the Non-Negative Matrix Factorization (NMF) method, superseding the conventional hierarchical clustering approach [11]. Utilizing the “NMF” function within the R package, we derived clusters from intrinsic tumor sample characteristics, generating associated biological correlation coefficients. Survival trajectories for groups C1 and C2 were assessed and juxtaposed against the conventional categorizations within the tumor microenvironment.

### Profiling of tumor microenvironment infiltrating cells

The depiction of the tumor’s immune milieu was constructed from the prevalence of nine unique immune and stromal cell subsets. These subsets include macrophages, dendritic cells, neutrophils, natural killer (NK) cells, CD4+ T cells, CD8+ T cells, B cells, fibroblasts, and endothelial cells. The presence of infiltrative cells was determined by contrasting scores across two categories, rooted in the cellular constituents of the microenvironment.

### Creation of senescence-related genes signature

Following the deployment of univariate Cox regression, genes associated with senescence were delineated. To mitigate potential overfitting, these genes underwent further analyses via lasso regression and subsequent multivariate Cox regression. In tandem, an examination of the genes’ prognostic attributes was conducted. The risk score for individual samples was ascertained through the formula “RiskScore = eSi(Coefi * Expi)” [12]. Using the median of these risk scores as a demarcation, samples were classified either within a high-risk stratum (HRG) or a low-risk stratum (LRG). Subsequent assessments included a review of the overall survival metrics for HRG and LRG. Distinct survival trajectories for HRG and LRG, in relation to the genes under observation, were separately charted [13].

### Nomogram formulation and verification

For the identification of prime prognostic markers, we executed receiver operating characteristic (ROC) evaluations on both risk scores and clinical indices for the initial, third, and quintennial years. Leveraging the “rms” and “regplot” tools within the R framework, a graphical representation was fashioned, and the model’s alignment was appraised via a calibration trajectory.

### Categorization of senescence-associated genes based on functionality

For the functional classification of genes within both the HRG and LRG, we utilized Gene set enrichment analysis (GSEA) [14]. The eight most pronounced outcomes (P < 0.05) were then visualized for further study.

### Evaluation of risk characteristics and clinical variables

The distribution pattern of clinical attributes within the HRG and LRG was outlined, and the proportion of patients within the clinical variable subsets were exhibited.

### Procurement and analysis of epigenetic mutation data

Relevant somatic alteration data was sourced from the TCGA–BLCA repository. We enumerated the twenty most commonly occurring genes exhibiting somatic deviations. Grounded on TMB risk and the bifurcation of samples into HRG and LRG, patients were classified into four primary cohorts. The survival likelihood for each cohort was assessed independently, leading to the generation of respective survival trajectories.

### Exploration of the relationship between risk scores and immune infiltration

To delineate the association between risk scores and immune infiltrating cells, we implemented tools such as XCELL, TIMER, QUANTISEQ, MCP COUNTER, EPIC, CIBERSORT, and CIBERSORT-ABS [15].

### Gene set variation analysis (GSVA)

We used the KEGG database for the analysis of the main gene pathway, evaluating the activation status of its signature and metabolic pathways [16]. Each sample underwent GSVA normalization for each gene set, allowing us to determine the relative activity of pathways, immune markers, and immune checkpoints.

### Predicting patient response to immunotherapy and chemotherapy

Genes pertinent to immune checkpoint modulation were pinpointed, and their expression dynamics were scrutinized in relation to the risk score. Leveraging the Immunophenoscore [17], we gauged the immunogenicity of tumors across both high and low-risk groups based on these gene profiles.

Drawing from TCGA–BLCA datasets, we structured a cellular expression evolutionary diagram. To probe into the genomic susceptibility of tumors to drugs, we employed the “pRRophetic” toolset within R, enabling insights into tissue responsiveness to diverse therapeutic agents [12, 18].

### Statistical methods

For multigroup data analyses, we invoked the Kruskal–Wallis evaluation, whereas binary group comparisons were facilitated using the Wilcoxon assessment. The Kaplan–Meier log-rank approach was chosen to assess survival trajectories. To discern associations between risk score divisions and somatic mutation occurrences, a chi-square assessment was conducted. Meanwhile, the Spearman methodology facilitated the determination of correlation indices. Results procured from the CIBERSORT methodology with a P-value below 0.05 underwent subsequent scrutiny. The benchmark for deeming statistical significance was earmarked at P < 0.05. All statistical undertakings were orchestrated within the R computational environment.

## Results

### Batch effect mitigation and data normalization

**Fig. 1:**
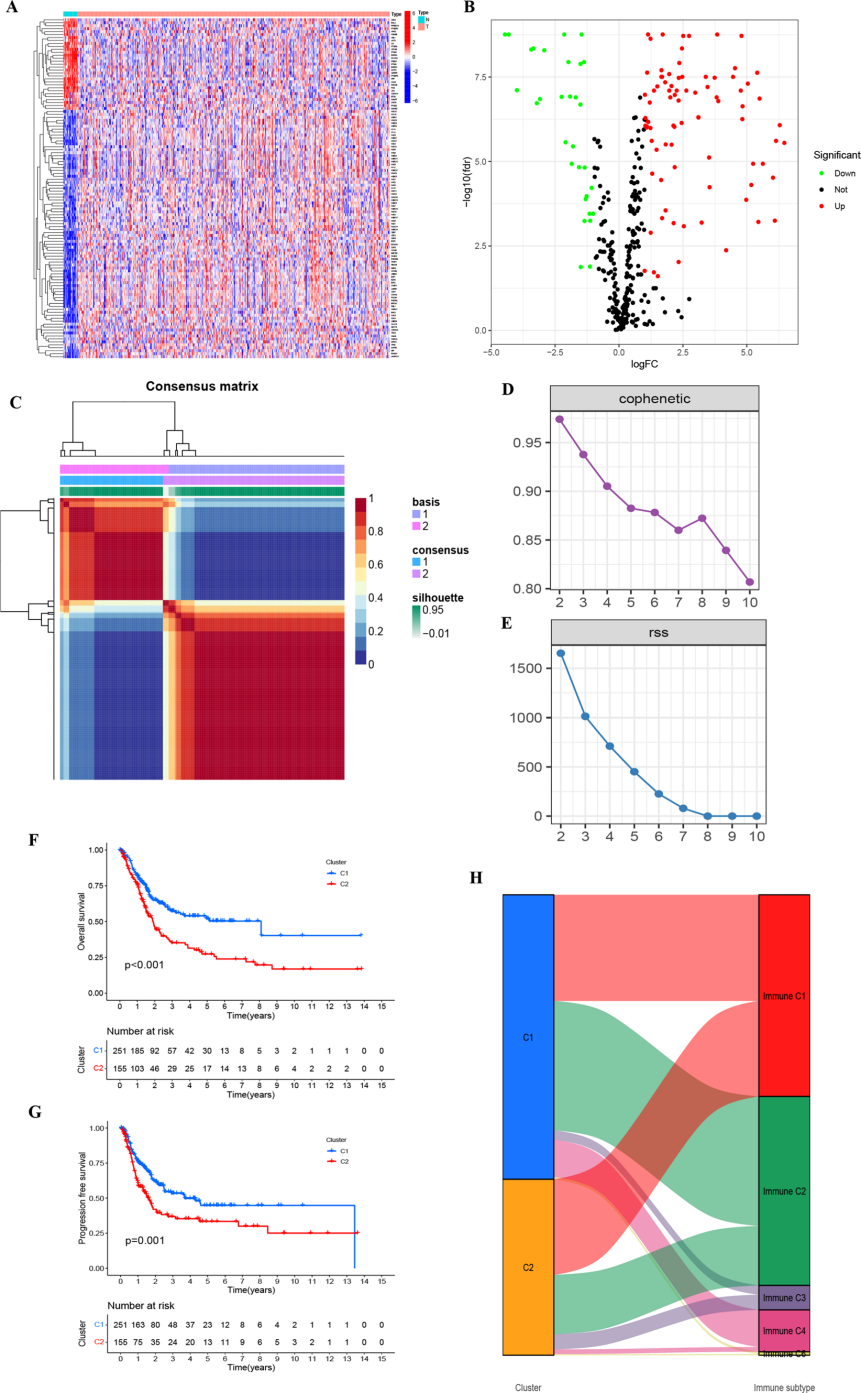
**A** Illustrates the expression levels of senescence-associated genes across various TCGA–BLCA samples. **B** Volcano plot depicting differentially expressed genes. **C** Clustering map derived through NMF algorithm. **D**, **E** Assessment of performance and stability pertaining to clusters through multiple methods. **F** Notable variance in overall survival observed between C1 and C2. **G** Significant disparity in progression-free survival between C1 and C2. **H** Distribution of C1 and C2 across immune molecule subtypes C1–C6

From the TCGA–BLCA dataset, we secured 406 neoplastic tissue specimens, coupled with their clinical data, and an additional 19 typical tissue specimens. Following the rectification of batch discrepancies, we instituted data standardization procedures. Setting our criteria at FDR < 0.05 and an absolute value of log2 (FC) surpassing 1, we discerned 115 genes with differential expression (Fig. 1A, Table S2). Figure 1B depicts a volcano plot illustrating all DEGs based on our selection criteria, emphasizing their significance and expression changes.

### Application of NMF in analyzing molecular subtypes of senescence-associated genes

**Fig. 2:**
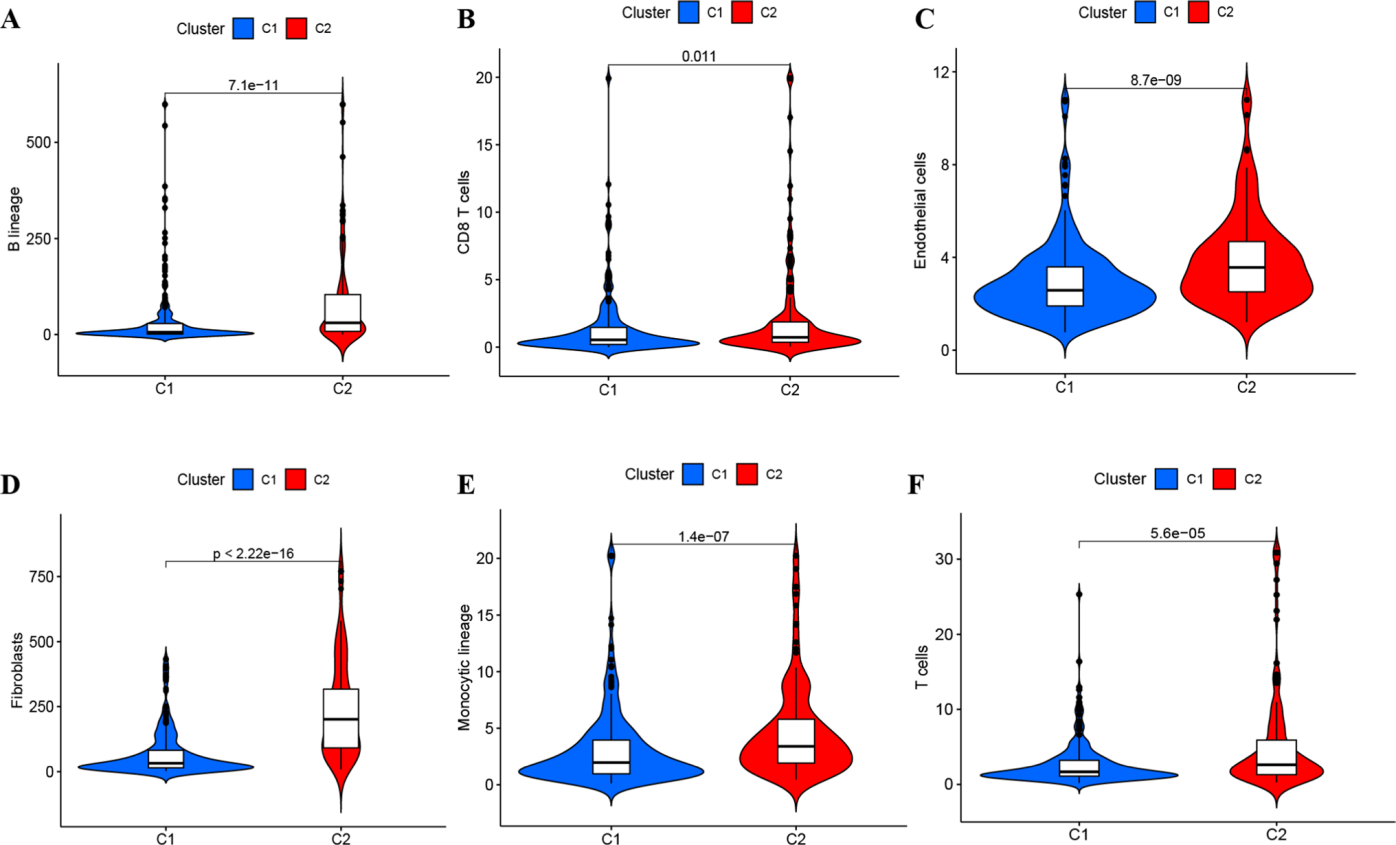
**A**–**G** Highlights the differential manifestations of C1 and C2 at the immune cellular level within the tumor microenvironment

Transcriptomic data from TCGA–BLCA were analyzed using the NMF clustering technique, with cophenetic and RSS parameters directing the process to ensure stability and optimal clustering outcomes. The optimal cluster number was identified as K = 2, facilitating the segregation into two distinct subgroups, namely C1 and C2 (Fig. 1C–E, Table S3). When evaluating survival curves, C1 samples manifested enhanced survival probabilities in contrast to C2 samples (Fig. 1F, G). We categorize tumor samples into six immune subtypes to elucidate the tumor microenvironment’s complexity: wound healing (C1), IFN-γ dominant (C2), inflammatory (C3), lymphocyte depleted (C4), immunologically quiet (C5), and TGF-β dominant (C6). Such analysis also illuminated the correlation between C1 and C2 clusters and the conventional immune classifications (Immune C1–C6) (Fig. 1H) [19]. Notably, marked variations in the composition of immune and stromal cell infiltrations between C1 and C2 were discerned (Fig. 2A–F). This underscores that the molecular categories arising from clustering based on senescence-associated genes seem intricately connected to diverse cell infiltrations within the tumor milieu.

### Construction and validation of prognostic models for senescence-associated genes

**Fig. 3:**
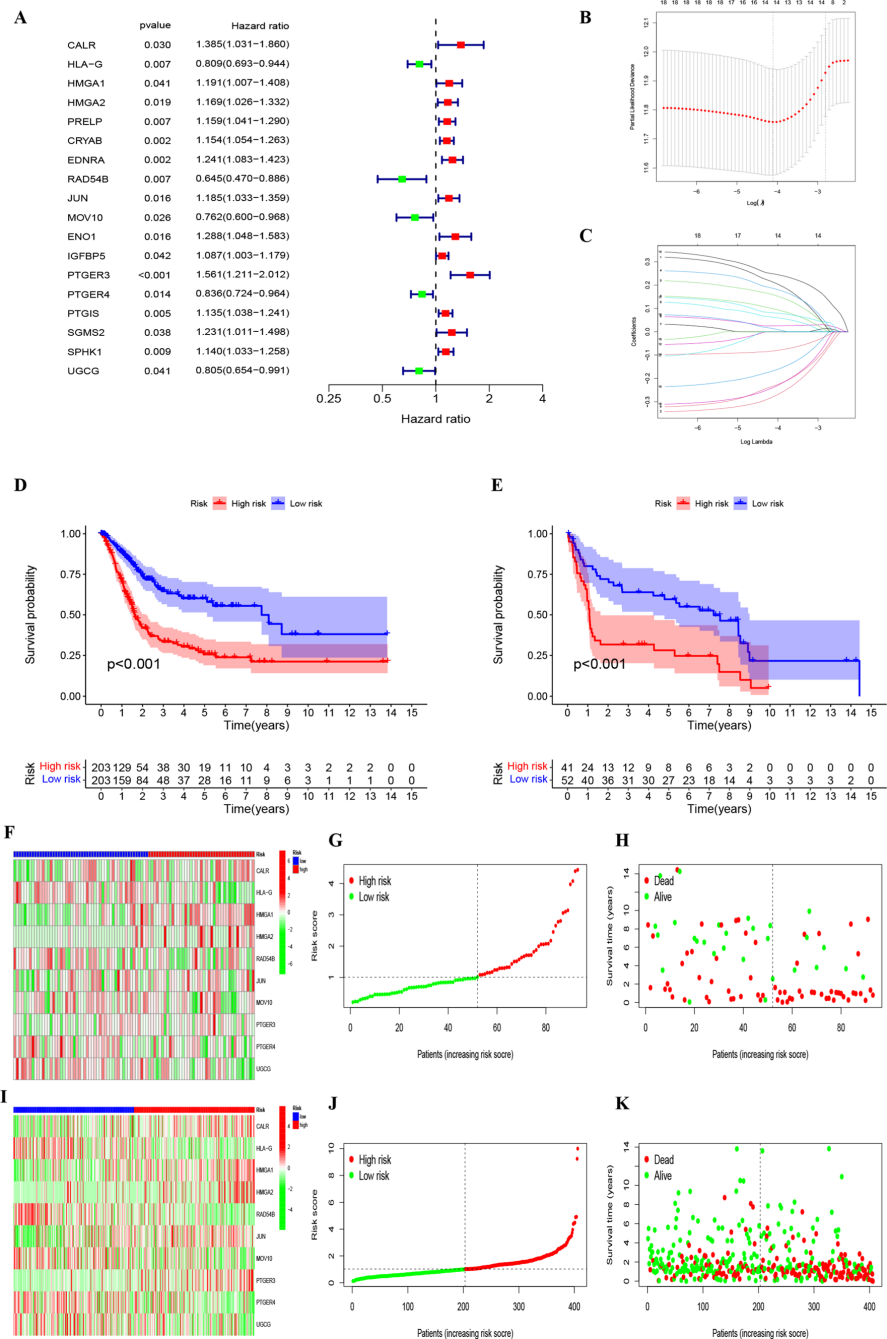
**A** Forest plot revealing the Univariate Cox regression analysis results pertaining to 18 senescence-associated genes and overall survival. **B**, **C** LASSO coefficient profiles for the 18 genes, characterized by vertical lines corresponding to tenfold cross-validation values. Ten cross-validations employed for tuning parameter selection in lasso regression, with vertical lines indicating the optimal data based on the minimum criterion and 1 standard error criterion. The left vertical line represents the 14 genes finally identified. **D** Kaplan–Meier curve analysis conducted on the TCGA database, highlighting the survival disparity between the HRG and LRG. **E** Kaplan–Meier curve analysis performed on the GEO database, indicating survival differences between HRG and LRG. **F** Validation of prognostic risk scores in the GEO cohort. **G** Risk score distribution for the polygenic model in the GEO cohort. **H** Survival status and duration for BLCA patients in the GEO cohort. **I** Validation of prognostic risk scores in the TCGA cohort. **J** Risk score distribution for the polygenic model in the TCGA cohort. **K** Survival status and duration for BLCA patients in the TCGA cohort

Starting with 115 differentially expressed genes, identified by stringent criteria of an absolute log2 fold change > 1 and an adjusted P-value (FDR) < 0.05, we further refined our analysis to focus on their prognostic significance. Through the application of univariate Cox regression, 18 genes of pronounced prognostic relevance were discerned (P < 0.05, Table S4, Fig. 3A). To counteract potential overfitting, these genes underwent lasso and multivariate COX regression evaluations, pinpointing ten pivotal genes (CALR, HLA-G, HMGA1, HMGA2, RAD54B, JUN, MOV10, PTGER3, PTGER4, UGCG) in BLCA with diagnostic significance (Fig. 3B, C). The formulation for RiskScore was: (0.3286 * CALR) − (0.3575 * HLA-G) + (0.2063*HMGA1) + (0.2359 * HMGA2) − (0.3606 * RAD54B) + (0.1659 * JUN) − (0.2371 * MOV10) + (0.3914 * PTGER3) − (0.1126 * PTGER4) − (0.3114 * UGCG). The coefficients for each gene in the RiskScore formula were derived using a multivariate Cox proportional hazards regression model. This model assesses the independent effect of each gene’s expression level on patient survival, assigning a weighted coefficient that quantifies its impact, as exemplified by the coefficient 0.3286 for CALR.

Using the median of the derived risk scores, tumor specimens were delineated into a high-risk category (HRG) and a counterpart low-risk category (LRG). Subsequent survival evaluations indicated superior outcomes for the LRG (Fig. 3D, E). Similar RiskScore distribution trends were observed in both GEO and TCGA cohorts, as illustrated in Fig. 3F–K. This consistency across different datasets reinforces the robustness and generalizability of our RiskScore as a prognostic tool in bladder cancer.

### Development of a risk nomogram

**Fig. 4:**
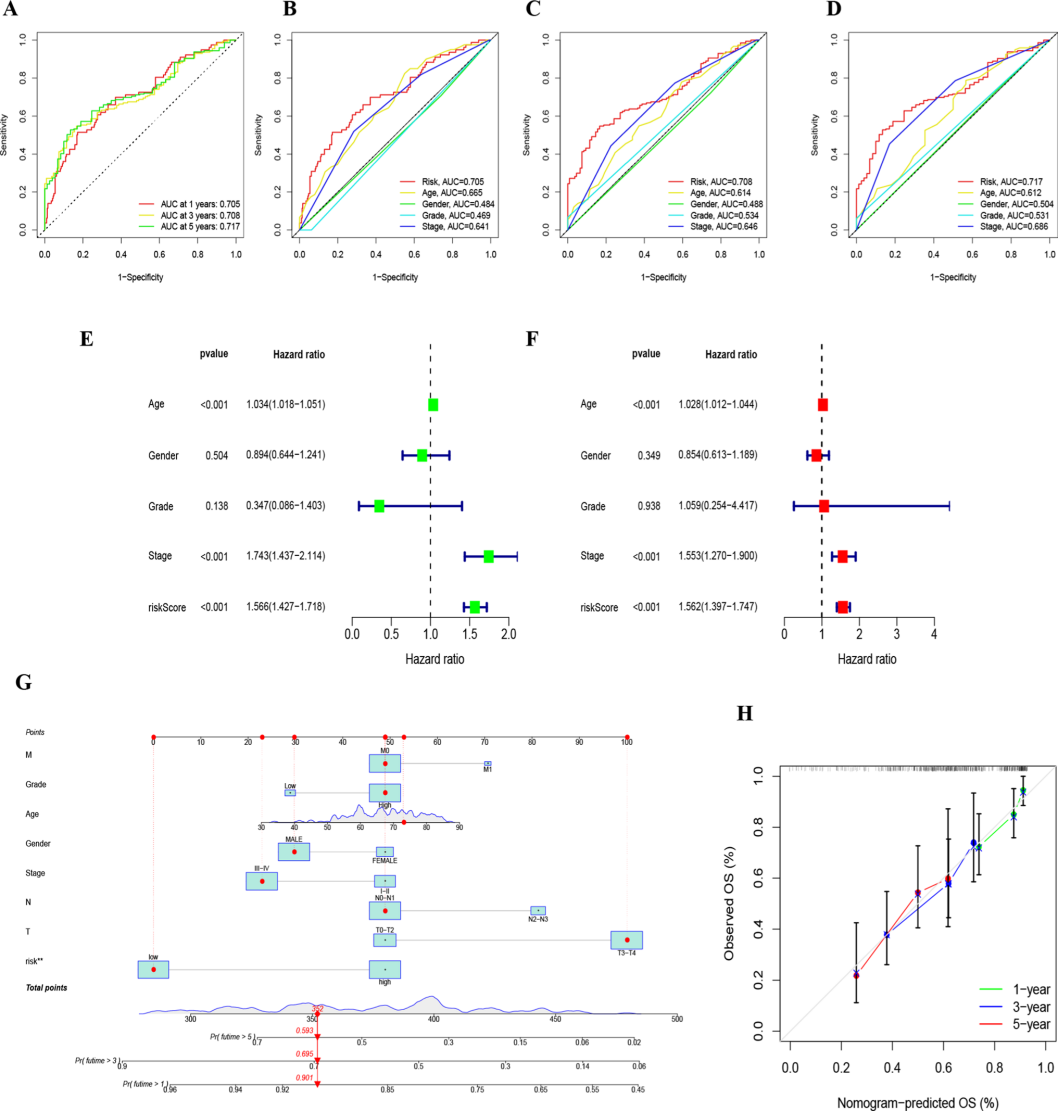
**A**–**D** ROC analysis forecasting 1-year, 3-year, and 5-year overall survival. **E** Univariate Cox regression analysis results of overall survival. **F** Multivariate Cox regression analysis results of overall survival. **G** Nomograph predicting survival outcomes. **H** Calibration curve for the Nomograph

ROC curve analyses were executed, producing areas under the curve values of 0.705, 0.708, and 0.717, highlighting noteworthy prognostic potential (Fig. 4A). An encompassing evaluation over 5 years, factoring in risk score, age, gender, tumor gradation, and clinicopathological staging (Fig. 4B–D), posited the risk score as a paramount predictor in comparison to other clinical parameters. Both univariate and multivariate Cox regression investigations validated age, disease stage, and risk score as standalone predictors of prognosis in BLCA patients (Fig. 4E, F). Leveraging the deduced risk scores and clinical metrics, a predictive nomogram was constructed to forecast survival probabilities at 1, 3, and 5-year intervals (Fig. 4G). The fidelity of this model was accentuated by the accompanying calibration plots (Fig. 4H) [20, 21].

### Examination of the function of senescence-associated genes

**Fig. 5:**
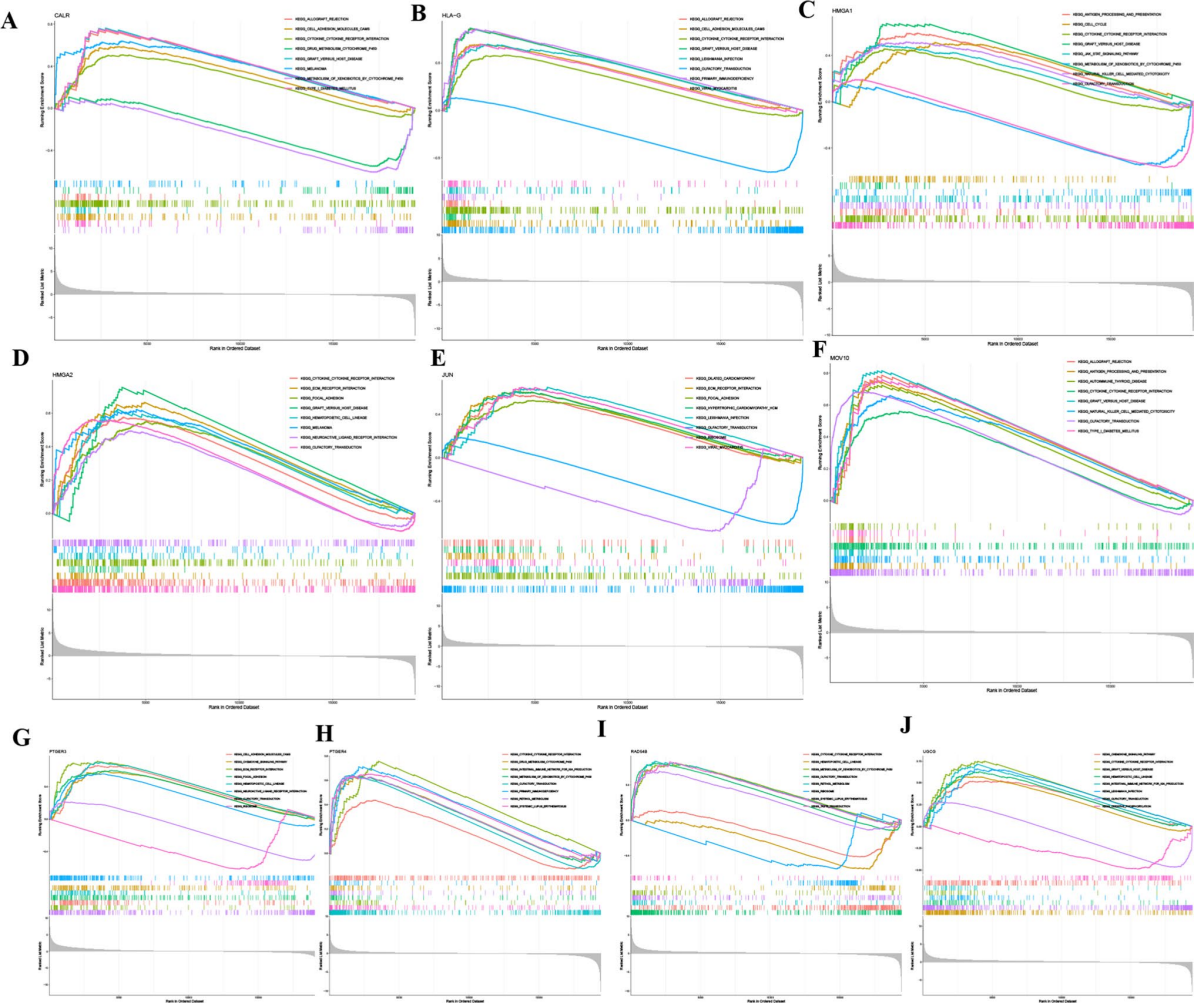
Functional enrichment analysis. **A** Enrichment gene set for samples expressing high levels of CALR in KEGG. **B** Enrichment gene set for samples expressing high levels of HLA-G in KEGG. **C** Enrichment gene set for samples expressing high levels of HMGA1 in KEGG. **D** Enrichment gene set for samples expressing high levels of HMGA2 in KEGG. **E** Enrichment gene set for samples expressing high levels of JUN in KEGG. **F** Enrichment gene set for samples expressing high levels of MOV10 in KEGG. **G** Enrichment gene set for samples expressing high levels of PTGER3 in KEGG. **H** Enrichment gene set for samples expressing high levels of PTGER4 in KEGG. **I** Enriched gene set for RAD54B high-expression samples in KEGG. **J** Enriched gene set for UGCG high-expression samples in KEGG

Through Gene Set Enrichment Analysis (GSEA), we explored the functional roles associated with the elevated and diminished expressions of the ten pivotal genes. Using KEGG enrichment evaluation, it was discerned that enhanced expression levels of HMGA2 and MOV10 were affiliated with pathways such as Versus-Host-Disease and Receptor-Interaction, among others. Augmentation of the Olfactory-Transduction pathway appeared to influence the surge in RAD54B expression. Enhanced expressions of PTGER3 and PTGER4 were speculated to correlate with the activation of pathways like Neuroactive-Ligand-Receptor-Interaction and other relevant signaling processes (Fig. 5A–J).

### Connection of risk factors with clinicopathological parameters

**Fig. 6:**
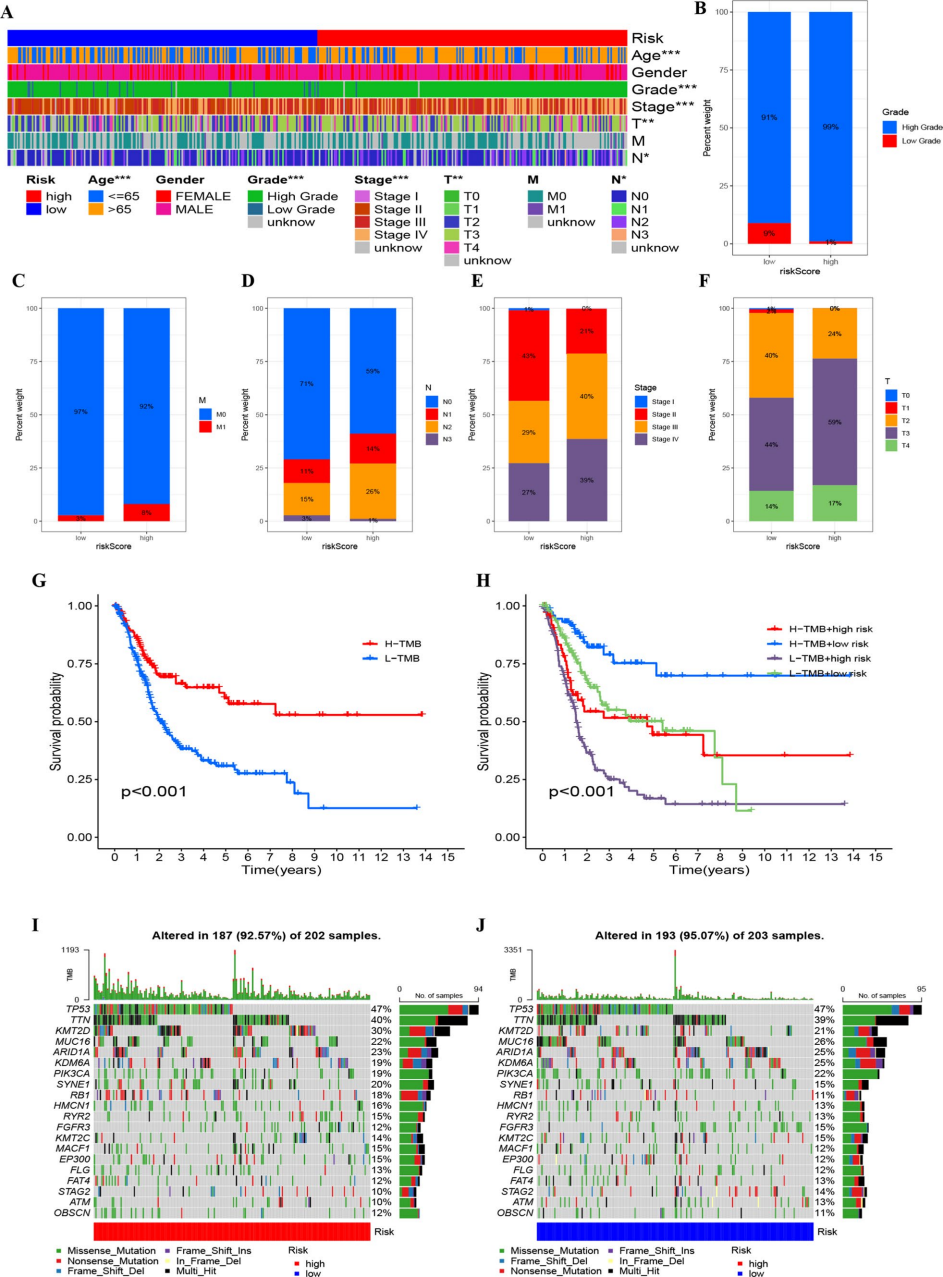
**A** Heatmap depicting the distribution of clinical characteristics and associated risk scores within each sample. Incidence of clinical variable subtypes in high and low risk score groups. **B** WHO grade, **C** distant metastasis M, **D** lymph node involvement N, **E** stage, **F** T, **G** Kaplan–Meier curve for TMB high and low groups. **H** Kaplan–Meier curve for patients within TMB high and low risk score groups. **I** Creation of HRG oncoPrint. **J** Creation of low-risk score oncoPrint

Clinicopathological parameters were displayed for both the HRG and LRG (Fig. 6A), and proportional diagrams were plotted to depict the clinical variables in both groups (Fig. 6B–F), unveiling significant differences between the HRG and LRG. Notably, the high-risk group was more frequently associated with advanced tumor stages (T3–T4), metastatic involvement (M1), and lymph node positivity (N+), as detailed in the proportional comparisons of Fig. 6C–F. These trends underscore the prognostic implications of the risk score in correlating with more aggressive disease features.

### Tumor mutational burden related clinical features

The analysis of survival curves revealed an extended overall survival for patients possessing elevated TMB (P < 0.001, Fig. 6G). By integrating both TMB and risk evaluations, patients were stratified into four distinct categories. There were marked prognostic variations between the HRG and LRG within both high and low TMB classifications (P < 0.001, Fig. 6H), suggesting the predictive prowess of the risk metric in gauging the efficacy of immunotherapy.

To delve into the relationship between risk evaluations and genetic aberrations, we delineated waterfall plots highlighting the top 20 frequently mutated genes (Fig. 6I, J). A survey of significant gene alterations revealed a predominant mutation rate for KMT2D in the HRG (30% vs 21%) and for MUC16 in the LRG (22% vs 26%). Such insights hold potential value for integrating senescence in BLCA therapeutic strategies [22, 23].

### Exploration of risk characteristics within the immune microenvironment

**Fig. 7:**
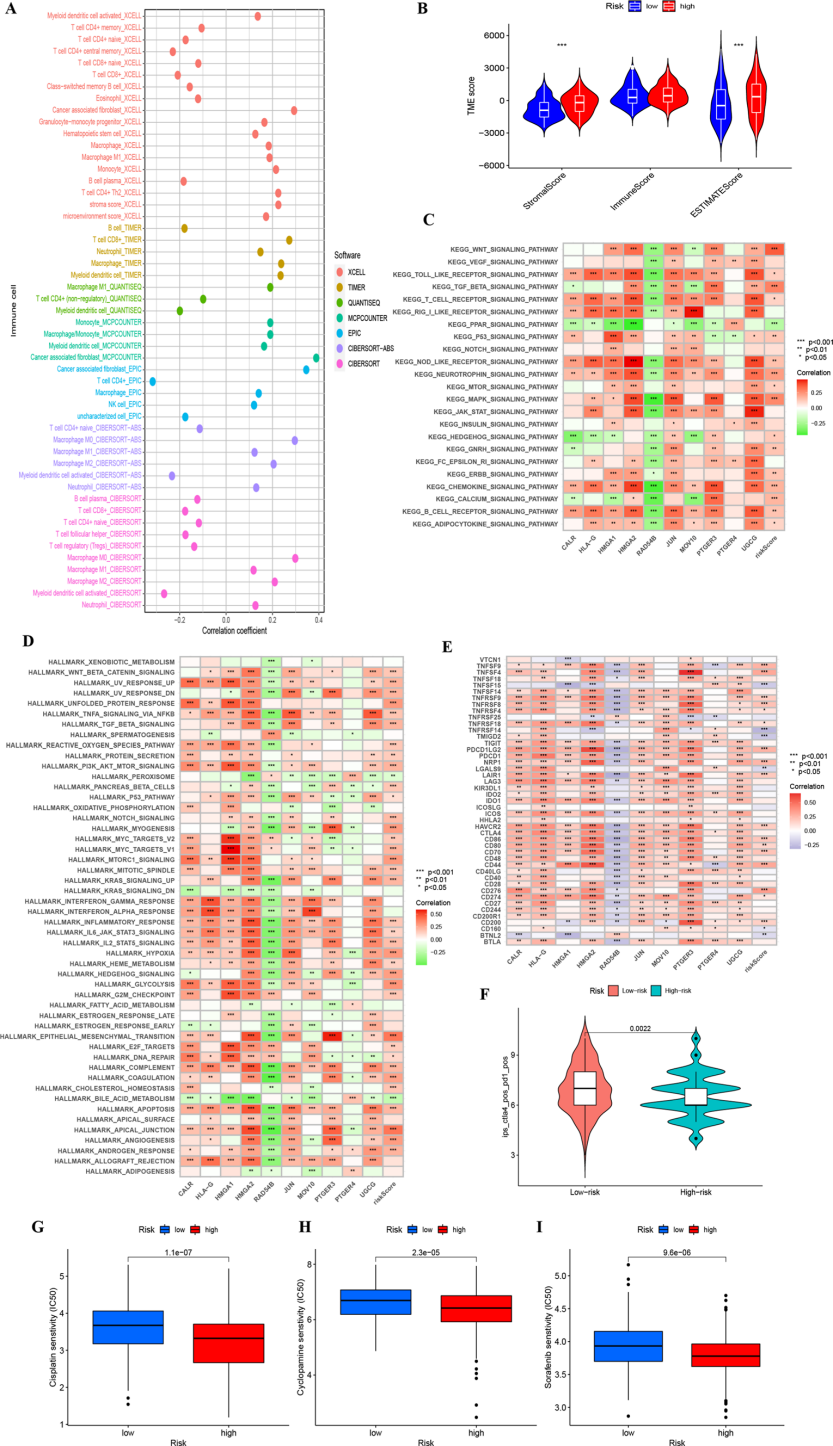
**A** Spearman analysis on the correlation between HRG patients and tumor-infiltrating immune cells. **B** ESTIMATE analysis of the TME score for HRG and LRG. **C** Correlation between KEGG’s representative pathway and risk score. **D** Correlation between Hallmark’s representative pathway and risk score. **E** Correlation between gene expression levels at immune checkpoints and risk scores. **F** Distribution chart for IPS scores. **G** Sensitivity analysis of cisplatin in high and low risk score groups. **H** Sensitivity analysis of cyclopamine in high and low risk score groups. **I** Sensitivity analysis of sorafenib in high and low risk score groups

We further explored the association between the risk score and immune cell infiltration employing seven unique analytical approaches (Fig. 7A). we observed notable variances across databases, including discrepancies in CD8+ and CD4+ T cell prevalence. While the EPIC analysis highlighted the predominance of CD4+ T cells in the tumor microenvironment, contrasting findings were observed when analyzing data from other platforms, where CD8+ T cells also showed significant infiltration patterns. This discrepancy underscores the complexity of the tumor immune landscape, suggesting that both cell types play crucial roles in bladder cancer’s immune contexture. The ESTIMATE [24] assessment revealed an ascending trajectory in both stromal and estimate scores across the HRG and LRG groups (Fig. 7B).

### Enrichment examination of biological functions and signal pathways

Through gene set variation analysis centered on the ten pivotal genes, we deepened our understanding of the biological functions associated with distinct risk classifications (Fig. 7C, D). Observations highlighted an augmented activation of the PPAR signaling pathway in subjects within the LRG, while pathways like the RIG-I-Like-Receptor and NOD-Like-Receptor exhibited enhanced activity in those categorized as HRG.

### Prognostication of patient outcomes following immunotherapy

Subsequent detailed analysis pinpointed 47 genes associated with checkpoint blockade. The inclusion of immune-related genes in our senescence-focused analysis is predicated on the emerging evidence that senescence can influence the tumor immune microenvironment. To elucidate this relationship, we specifically curated a set of immune-related genes known to interact with senescent cells or pathways, thus providing a comprehensive view of how senescence may modulate immune evasion and therapy response. The majority manifested a negative association with the risk score and were deemed significant (Fig. 7E). Prognostic models presented diminished IPS scores for those in the high-risk category, hinting that such patients might not be optimal recipients for PD-1 and CTLA-4 immunotherapies (Fig. 7F). This robust data suggests a noteworthy linkage between risk scores and immunotherapy responses, endorsing its potential use in forecasting prognosis.

### Forecasting chemotherapeutic responsiveness

Through the application of the pRRophetic algorithm, we assessed the IC50 values for three therapeutic agents (cisplatin, cyclopamine, sorafenib) among BLCA patients. Elevated IC50 values were observed for cisplatin, cyclopamine, and sorafenib within the LRG (P < 0.001), indicating a potential diminished drug responsiveness (P < 0.001) (Fig. 7G–I). Such findings may inform tailored drug recommendations for patients based on their specific risk profiles.

## Discussion

Our research delved profoundly into understanding the prognostic ramifications of genes associated with senescence in BLCA. Through this endeavor, we pinpointed ten cardinal genes with pronounced prognostic relevance for individuals diagnosed with BLCA. This endeavor has enriched our comprehension of BLCA’s underlying molecular dynamics and accentuated the significance of these genes as potential prognostic indicators. It’s pertinent to highlight that the prominence of these senescence-associated genes in BLCA is attributed to their regulatory influence on cellular dynamics, thereby modulating disease trajectory and patient prognoses [25, 26].

To render these discerned genes into a practical diagnostic asset, we amalgamated them to formulate a risk score [27, 28]. Our findings underscore the efficacy of this devised risk score in bifurcating BLCA patients into cohorts of heightened or diminished risk, furnishing clinicians with a tool to refine prognostic evaluations and therapeutic choices. To ascertain the model’s resilience and precision, it underwent verification using both the TCGA–BLCA and GEO databases, consistently affirming its predictions across both datasets. Crucially, individuals grouped within the elevated-risk segment manifested shorter survival times compared to the diminished-risk group, emphasizing the practical utility and promise of our senescence-associated gene-centric model for optimizing BLCA patient care. To address the clinical applicability of our risk scoring model, we propose integrating it with current diagnostic workflows, enhancing patient stratification for tailored treatments. Future studies should focus on validating the model in clinical settings and identifying potential barriers to its adoption, such as the need for high-throughput genetic testing facilities.

Delving deeper into the intricacies of the identified genes has provided us a clearer picture of their multifarious biological roles and intertwined signaling pathways. It’s noteworthy that augmented expression levels of certain genes, namely HMGA2 [29] and MOV10, were seamlessly interlaced with an array of sophisticated biological undertakings and signaling cascades, encompassing aspects such as cellular senescence, immune modulation, and tumorigenesis. The extensive scope of their function insinuates a multi-dimensional impact of these genes on the trajectory and perhaps onset of BLCA. In light of the study by Luo et al. [9], which explored the prognostic value of senescence-related genes in bladder cancer, our research builds upon and extends these findings. While Luo et al. identified crucial genes within this context, our analysis introduces additional senescence-associated genes and elaborates on their mechanistic implications in tumor behavior and immune interactions. This comparative acknowledgment not only situates our work within the existing research paradigm but also showcases our novel contributions towards refining the prognostic utility of senescence-related genes in bladder cancer. The integration of our risk score model, predicated on an expanded gene set, offers a promising avenue for enhancing patient stratification and treatment decision-making.

Moreover, the constructed risk score model depicted strong correlations with diverse clinicopathological variables, thereby reinforcing its applicability in a clinical scenario. A salient observation from our investigation was the pronounced interplay between the risk score and the Tumor Mutational Burden (TMB). Given TMB’s emerging potential as a predictor of immunotherapy responsiveness in BLCA, such an interconnection could have profound implications for tailoring therapeutic regimens [30, 31]. Furthermore, our data uncovered an intriguing nexus between the risk scores and the nuances of the immune landscape. Specifically, a counteractive relationship between CD8+ T cell abundance and risk scores was discerned, suggesting a nuanced crosstalk between cellular senescence, immune dynamics, and cancerous evolution in BLCA. The documented elevated stromal and immune indices in high-risk cohorts underscore the pivotal influence of the immune milieu on BLCA’s clinical outlook.

To encapsulate, our research illuminates the cardinal influence of genes associated with senescence in the diagnostic and therapeutic paradigms of BLCA. The correlations unraveled, encompassing clinical trajectories, TMB, and immune contexts, potentially signal a transition towards bespoke treatment strategies. These profound insights provide a robust foundation for ensuing studies, with an ambition to elucidate the multifaceted implications of genes tied to senescence in the realm of BLCA and beyond.

## Conclusion

We constructed a predictive framework grounded on ten genes linked to senescence, along with clinical risk determinants, proficient in forecasting survival outcomes, immune reactions, and chemotherapeutic responses in BLCA. Multiple layers of validation underscore its efficacy and promise as a credible instrument for prognostication in BLCA.

## Limitations

(1) Dataset size and composition: our analysis was primarily conducted using data sourced from the TCGA and GEO repositories. While these databases are extensive and provide a rich foundation for research, the size and diversity of the datasets, especially the relatively smaller number of normal tissue samples compared to neoplastic specimens, may limit the generalizability of our findings across different populations and bladder cancer subtypes. (2) validation studies: although we have validated our prognostic models using available datasets from GEO, independent validation studies, particularly prospective studies, are required to further establish the robustness and clinical applicability of our senescence-associated gene signatures in bladder cancer prognosis.

### Supplementary Information

Below is the link to the electronic supplementary material.

Supplementary file 1 (TXT 3 KB)

Supplementary file 2 (TXT 11 KB)

Supplementary file 3 (TXT 7 KB)

Supplementary file 4 (TXT 1 KB)

## Data Availability

The datasets analyzed during the current study are publicly available in the following repositories: The Cancer Genome Atlas (TCGA) data for bladder cancer (BLCA) are available through the Genomic Data Commons (GDC) Data Portal. These data can be accessed at https://portal.gdc.cancer.gov/, by selecting the “Bladder Urothelial Carcinoma (BLCA)” project. Gene Expression Omnibus (GEO) datasets utilized in this study can be found under accession number GSE31684. The dataset can be accessed directly at https://www.ncbi.nlm.nih.gov/geo/query/acc.cgi?acc=GSE31684. These repositories provide comprehensive access to the raw and processed data supporting the results and analyses presented in this article. Users are encouraged to refer to the respective repository guidelines on data usage policies and procedures for accessing and utilizing the datasets.
